# How does plant chemodiversity evolve? Testing five hypotheses in one population genetic model

**DOI:** 10.1111/nph.20096

**Published:** 2024-09-05

**Authors:** Meike J. Wittmann, Andrea Bräutigam

**Affiliations:** ^1^ Faculty of Biology, Theoretical Biology Bielefeld University Universitätsstraße 25 33615 Bielefeld Germany; ^2^ Joint Institute for Individualisation in a Changing Environment (JICE) University of Münster and Bielefeld University 33615 Bielefeld Germany; ^3^ Faculty of Biology, Computational Biology Bielefeld University Universitätsstraße 25 33615 Bielefeld Germany; ^4^ Center for Biotechnology Bielefeld University Universitätsstraße 25 33615 Bielefeld Germany

**Keywords:** interaction diversity hypothesis, mathematical model, phytochemical diversity, plant–herbivore interactions, screening hypothesis, synergy hypothesis

## Abstract

- Plant chemodiversity, the diversity of plant‐specialized metabolites, is an important dimension of biodiversity. However, there are so far few mathematical models to test verbal hypotheses on how chemodiversity evolved. Here, we develop such a model to test predictions of five hypotheses: the ‘fluctuating selection hypothesis’, the ‘dominance reversal hypothesis’, the interaction diversity hypothesis, the synergy hypothesis, and the screening hypothesis.
- We build a population genetic model of a plant population attacked by herbivore species whose occurrence fluctuates over time. We study the model using mathematical analysis and individual‐based simulations.
- As predicted by the ‘dominance reversal hypothesis’, chemodiversity can be maintained if alleles conferring a defense metabolite are dominant with respect to the benefits, but recessive with respect to costs. However, even smaller changes in dominance can maintain polymorphism. Moreover, our results underpin and elaborate predictions of the synergy and interaction diversity hypotheses, and, to the extent that our model can address it, the screening hypotheses. By contrast, we found only partial support for the ‘fluctuating selection hypothesis’.
- In summary, we have developed a flexible model and tested various verbal models for the evolution of chemodiversity. Next, more mechanistic models are needed that explicitly consider the organization of metabolic pathways.

## Abstract 2

See also the Commentary on this article by Speed & Ruxton, **245**: 924–926.

## Introduction

Plants harbor an amazing diversity of so‐called specialized (or secondary) metabolites. These are not involved in basic functions like photosynthesis that are shared by (almost) all plants. Rather, different plant lineages have different sets of specialized metabolites to defend themselves against herbivores, attract pollinators, or fulfill other yet unknown functions (Huang & Dudareva, 2023). This ‘chemodiversity’ raises several evolutionary questions: Why are there so many metabolites? And why do individuals differ qualitatively and quantitatively in the metabolites that they produce? That is, what maintains chemical polymorphism in populations?

Potential answers to such questions are offered by a number of hypotheses and verbal models (for reviews, see Moore *et al*., 2014; Wetzel & Whitehead, 2020; Thon *et al*., 2024). To test whether these verbal models ‘work’, that is whether the claimed predictions follow from the assumptions, and to find out what exact patterns of chemodiversity they predict, mathematical models, and computer simulations are needed (Servedio *et al*., 2014). Such models exist for genetic variation and for species diversity, but so far, there are few mathematical and simulation models for chemodiversity (reviewed in Thon *et al*., 2024). These include optimality models that predict investment in defenses based on resource constraints (e.g. Coley *et al*., 1985; Yamamura & Tsuji, 1995; Orrock *et al*., 2015), evolutionary game theory, and frequency‐dependent selection models that explain coexistence between defended and undefended plants based on direct and indirect interactions between plants in neighborhoods (e.g. Augner *et al*., 1991; Lankau, 2009; Sato *et al*., 2017), simulation models for plant–herbivore coevolution (Speed *et al*., 2015; Zu *et al*., 2020; see also Bass & Kessler, 2022; Zu *et al*., 2021), as well as differential‐equation models for eco‐evolutionary dynamics between plants, herbivores, and pollinators (McPeek *et al*., 2022). While some models specifically address variation in chemical traits, others more generally address absence or presence of defense traits, but could be also be applied to absence or presence of a single defense metabolite.

However, several important verbal hypotheses still lack underpinning by mathematical models. First, the interaction diversity hypothesis (Iason *et al*., 2011; Whitehead *et al*., 2021) posits that plants produce many different metabolites because they engage in many ecological interactions mediated by different metabolites. Second, the synergy hypothesis suggests that plants produce many metabolites because the antiherbivore effects of mixtures are often larger than expected based on adding the effects of individual metabolites (Richards *et al*., 2016). Third, the screening hypothesis (Jones & Firn, 1991; Firn & Jones, 2003) suggests that each new metabolite has only a small probability of having biological activity in a relevant ecological interaction, for example against a herbivore, forcing plants to produce a large number of metabolites to ‘find’ the few useful ones. Since the machinery for producing metabolites is costly, the screening hypothesis further predicts that plants should evolve grid‐like pathways with promiscuous enzymes that can efficiently produce a large diversity of metabolites.

Furthermore, although much of chemodiversity has a genetic basis, there is a surprising lack of population genetic models for chemodiversity. One powerful mechanism for the maintenance of genetic variation is temporally fluctuating selection where heterozygotes have the highest fitness in the geometric mean over time (called marginal overdominance), although they may not have the highest fitness at any particular time (e.g. see Haldane & Jayakar, 1963; Hedrick, 1976; Wittmann *et al*., 2017; Johnson *et al*., 2023). Since many insect herbivores fluctuate in abundance over time (Root, 1996; Stange *et al*., 2011; De‐la‐Cruz & Núñez‐Farfán, 2023), for example due to temperature variation between seasons or across years, fluctuating selection could maintain chemodiversity between individuals in a population. For example, if a metabolite repels generalist herbivores, but attracts specialist herbivores, a genetic polymorphism at an underlying locus could potentially be maintained by fluctuations in the presence of specialist and generalist herbivores (as suggested for the glucosinolate sinigrin by Lankau, 2007). However, this ‘fluctuating selection hypothesis’ has not been explored in a mathematical model.

Finally, we know from population genetic theory that the maintenance of polymorphism can strongly depend on patterns of genetic dominance. Dominance quantifies the phenotype or fitness of heterozygotes relative to homozygotes. Consider a locus with two alleles, 1 (presence) and 0 (absence), where ‘11’ homozygotes produce a metabolite, but ‘00’ homozygotes do not. The 1 allele would be called (partially) dominant if ‘10’ heterozygotes produce more than half of what 11 homozygotes produce and recessive if heterozygotes produce less than half. If a locus influences two or more phenotypes (pleiotropy), dominance coefficients can differ between phenotypes (Grieshop *et al*., 2024). Antagonistic pleiotropy with reversals of dominance across contexts can be a powerful mechanism for the maintenance of genetic variation (Rose, 1982; Hoekstra *et al*., 1985; Curtsinger *et al*., 1994; Wittmann *et al*., 2017; Connallon & Chenoweth, 2019; Grieshop *et al*., 2024). For example, in the context of chemodiversity, if the 1 allele confers herbivore resistance and is also dominant for herbivore resistance, but has costs which reduce fecundity and is recessive for fecundity, similar to the case of fluctuating selection, heterozygote advantage (overdominance) could emerge at the level of overall fitness such that polymorphism is maintained. So far, there are no models exploring this ‘dominance reversal hypothesis’ for chemodiversity. From crossing experiments, we know that specialized metabolites and plant defense traits often exhibit dominance (Kondra & Stefansson, 1970; Orians, 2000; van Dam & Baldwin, 2003; Olson‐Manning *et al*., 2013), although to our knowledge, nothing is known on dominance reversals.

To help fill these gaps in chemodiversity modeling, here we develop a population genetic modeling approach focused on the evolution of chemodiversity via presence–absence polymorphisms, that is polymorphisms where some individuals in the population produce a metabolite while others do not. We use this model to explore qualitatively how patterns of chemodiversity are affected by herbivore fluctuations, dominance patterns, herbivore numbers, and synergistic effects of metabolites. For each scenario, we will analytically (if possible) determine the potential for genetic polymorphism and use individual‐based simulations to quantify the expected total number of metabolites produced in the population (γ diversity), the average number of metabolites produced per individual (α diversity), and the average number of different metabolites between two individuals (β diversity). Thereby, we perform proof‐of‐concept tests for the dominance reversal hypothesis, the fluctuating selection hypothesis, the interaction diversity hypothesis and the synergy hypothesis. Additionally, we test whether plant populations with higher chemodiversity are better able to cope with a new herbivore, as suggested by the screening hypothesis.

## Model description

We assume a diploid, randomly mating plant population of constant size *N* (Table 1 for a parameter overview). Each time step corresponds to one plant generation time. Adult plants die with probability θ per time step and there is no seed bank. We focus on the case of nonoverlapping generations $\left(\theta =1\right),$ but also consider overlapping generations $\left(\theta <1\right).$ The plant population can be attacked by $n_{h}$ herbivores. Time is divided into phases of g generations. We assume that herbivore *i* is present and active with probability $p_{i}$ independently for each phase and independently from other herbivores. If herbivore fluctuations are at the same time scale as plant generations, for example annually for an annual plant, g=1. If g>1 (our default is g=5), herbivore fluctuations are slower than plant generations, as in annual herbivore fluctuations for a weed with multiple generations per year or changes in the herbivore community every few years for an annual plant.

**Table 1:** Parameters and their default values. Unless noted otherwise, the parameters are kept at their default values.

Parameter	Explanation	Default value
*N*	Population size	500
θ	Adult death probability per time step	1
$n_{h}$	Number of herbivores	1
*g*	Number of generations per phase	5
$p_{i}$	Probability that herbivore *i* is present and active during a particular phase	0.2
L	Number of loci responsible for metabolite production = maximum possible number of different metabolites	10
$m_{\mathrm{li}}$	Protective effect of metabolite *l* against herbivore *i*	1
$d_{a}$	Dominance for effect on herbivores	Always varied
$d_{c}$	Dominance for costs	Always varied
$b_{0}$	Baseline probability to escape herbivore	0
$a_{\text{half}}$	Half‐saturation parameter of the benefit function	0
*s*	Sensitivity parameter of the benefit function	1
*c*	Cost parameter	0.02
*u*	Mutation probability per allele copy per generation	0.0001

The plant genome has *L* loci responsible for the production of metabolites that affect herbivores. They could be either the genes coding for the biosynthetic enzymes or regulatory loci affecting such genes. The $L\times n_{h}$ matrix $M=\left(m_{\mathrm{li}}\right)$ specifies antiherbivore activity of a certain locus *l* against a certain herbivore species *i*. If $m_{\mathrm{li}}>0$, the metabolite acts as a repellent for herbivore *i*, if $m_{\mathrm{li}}=0$, it has no effect, and if $m_{\mathrm{li}}<0$, the metabolite attracts the herbivore. At each locus, there can be two alleles: 0 (absence, that is a deletion or a nonfunctional gene‐variant) and 1 (presence). That is, individuals can have three possible genotypes at each locus: 00 or 11 homozygote or 01 heterozygote/hemizygote.

For each individual and for each herbivore species *i*, we first determine what antiherbivore activity $a_{i}$ the individual has against herbivore *i*. We here assume that activities are additive across loci but that there can be dominance effects:

(Eqn 1) $$a_{i}=\sum_{l=1}^{L}m_{\mathrm{li}}z_{a,l}$$

where

(Eqn 2) $$z_{a,l}=\left\{\begin{array}{cc} 0 & \text{for genotype}\;00\;\mathrm{at}\;\text{locus}\;l\\ d_{a} & \text{for genotype}\;01\;\mathrm{at}\;\text{locus}\;l\\ 1 & \text{for genotype}\;11\;\mathrm{at}\;\text{locus}\;l \end{array}\right.$$

We assume that the ‘activity dominance’ $d_{a}$ is between 0 and 1 and for simplicity is constant across loci (though we relax this later). With $d_{a}=0$, heterozygotes do not benefit at all from the single copy of the presence allele, whereas with $d_{a}$, they enjoy the same protective effects as an individual with two copies of the presence allele.

**Fig. 1:**
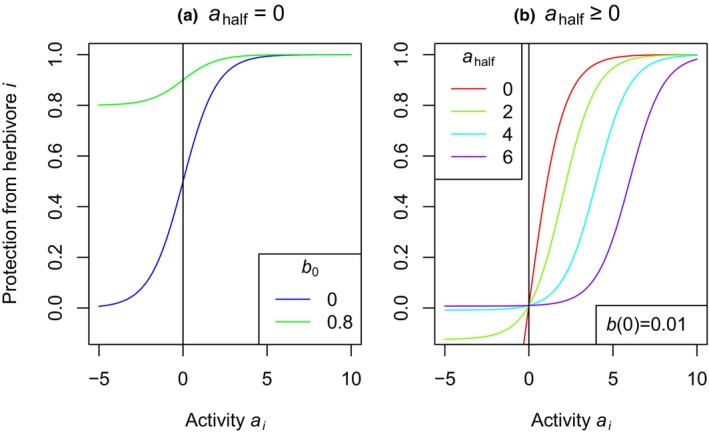
Benefit functions $b\left(a_{i}\right)$ used in this study. In (a), the green curve ($b_{0}=0.8$) represents a low herbivory scenario, whereas the blue curve ($b_{0}=0$) represents a high herbivory scenario. In (b), benefit functions with increasing half‐saturation parameter $a_{\text{half}}$ exhibit increasing synergy. The baseline probability to escape the herbivore $b_{0}$ for each scenario in (b) is chosen such that $b\left(0\right)=0.01$ in all four scenarios.

The activity *a* against a certain herbivore then affects fitness at times when the herbivore is present. We capture this in a benefit function $b\left(a\right)$. Different shapes of this function are possible. We here use a classical logistic benefit function

(Eqn 3) $$b\left(a\right)=b_{0}+\frac{1-b_{0}}{1+\exp\left(-s\cdot \left(a-a_{\text{half}}\right)\right)}$$

where $b_{0}$ is the baseline probability to escape the herbivore in the worst case that an individual is strongly attracting herbivores (large negative *a*). The parameter $b_{0}$ can be used to model different levels of herbivore pressure. $a_{\text{half}}$ is a half‐saturation parameter specifying at which activity level half of the possible protection is achieved, and s is the sensitivity of the protection to differences in the antiherbivore activity (Fig. 1a). The output $b\left(a\right)$ can be understood as the proportion of plant material not consumed by the herbivore or more abstractly as the proportion of fitness remaining after a generation where the respective herbivore is present. We here do not model cases where a plant is killed completely by herbivores before it has a chance to reproduce. Therefore, our model is most appropriate to plants attacked by relatively small herbivores such as insects rather than for cases where large mammal herbivores might kill entire plant individuals. We chose the specific shape of the benefit function because the protection from herbivores approaches 1 for very high activity levels and it is flexible enough to accommodate different shapes of benefit functions found in the literature (Wetzel *et al*., 2016; Pearse *et al*., 2018). With low $a_{\text{half}}$, the benefit function is saturating, at least over most of the positive range of activity levels while with high $a_{\text{half}}$, the benefits of two metabolites would be more than expected based on the sum of the benefits of each metabolite separately (Fig. 1b). This enables us to test predictions of the synergy hypothesis.

In addition to their protective benefits, metabolites also have costs. Here, we assume that fecundity is proportional to the cost function

(Eqn 4) $$f\left(z_{c}\right)=\exp\left(-c\cdot z_{c}\right)$$

where *c* is a cost parameter and

(Eqn 5) $$z_{c}=\sum_{l=1}^{L}z_{c,l}$$

is a ‘cost trait’ with

(Eqn 6) $$z_{c,l}=\left\{\begin{array}{cc} 0 & \text{for genotype}\;00\;\mathrm{at}\;\text{locus}\;l\\ d_{c} & \text{for genotype}\;01\;\mathrm{at}\;\text{locus}\;l\\ 1 & \text{for genotype}\;11\;\mathrm{at}\;\text{locus}\;l \end{array}\right.$$

where $d_{c}$ is the dominance parameter with respect to costs and is also assumed to be between 0 and 1. For the parameter settings we explore (Table 1), the most extreme reduction in fecundity is to $\exp\left(-0.02\cdot 10\right)=0.818$, that is a reduction by 18.2%, which is well within the range reported by Strauss *et al*. (2002) for direct costs (6–45% in studies with controlled genetic background, 8.7–73% in studies of natural populations). For moderate costs, our cost function is well approximated by a linear cost function (in this case 1−0.02⋅10=0.8). Most other nonlinear cost functions with moderate costs will also be well approximated by a linear function and thus by (5). The advantage of using the exponential over a linear function is that the exponential can never yield negative values even with more extreme cost settings.

An individual with activities $a_{1},\ldots ,a_{n_{h}}$ against the various herbivores and ‘cost trait’ $z_{c}$ then has the following fitness in generation *t*:

(Eqn 7) $$W_{t}=\left(\prod_{i=1,i\in H_{t}}^{n_{h}}b\left(a_{i}\right)\right)\cdot f\left(z_{c}\right)$$

where $H_{t}$ is the set of herbivores present in generation *t*. That is, we assume that all fitness components act multiplicatively.

Based on the death probability θ, the number of sites *F* that free up at the end of the time step is drawn from a binomial distribution with parameters *N* and θ. Individuals then reproduce in proportion to their fitness. That is, for each of the free sites, we draw one mother and one father individual (allowing incidental selfing) with weights proportional to their fitness. Such selection is called ‘soft’ because an individual's absolute fitness, that is number of offspring in the next time step, depends not only on its own trait but via competition for a limited number of places in the population also on the traits of its conspecifics (Bell *et al*., 2021). Mutations happen with probability *u* per generation per allele copy and then change an allele into the respective other allele. Finally, *F* adults are randomly killed to complete the time step.

### Mathematical analysis

Our first goal is to find the optimum number of 1 alleles (corresponding to extra metabolites, or upregulation of metabolites) for an individual. The long‐term fate of alleles and polymorphisms in a temporally variable environment with nonoverlapping generations depends on geometric mean fitness over time (Haldane & Jayakar, 1963), here

(Eqn 8) $$\tilde{W}=\left(\prod_{i=1}^{n_{h}}b{\left(a_{i}\right)}^{p_{i}}\right)\cdot f\left(z_{c}\right)$$

which does not depend on the length *g* of phases, their ordering, or autocorrelation effects (Gillespie, 1973). We use (8) to find the homozygous genotype (which has either 00 or 11 at each locus) with the highest fitness.

Next, we investigate whether and when there is scope for genetic polymorphism and thus also for variation in the chemical composition between individuals. For this, we use an invasion analysis similar to that in Wittmann *et al*. (2017). Such invasion arguments are also used in game theoretical or frequency‐dependent selection models (Augner *et al*., 1991; Sato *et al*., 2017), but usually under the explicit or implicit assumption of haploidy and asexual reproduction, whereas here, we consider sexually reproducing diploids and thus have to consider heterozygotes and dominance.

Given a fully monomorphic ‘resident’ population where all individuals have the same multi‐locus genotype and are homozygous at all loci, we determine whether or not a mutant with the respective other allele at a certain locus can invade (i.e. increase in frequency in the long run when it starts out at very low frequency). Since we assume random mating, almost all copies of the rare allele are in heterozygotes and the number of heterozygotes is $N\cdot 2x\cdot \left(1-x\right)\approx 2\mathrm{Nx}$, where *x* is the frequency of the rare allele. The rare allele increases in frequency if the number of heterozygotes increases in frequency. If $x_{t}$ is the allele frequency at time *t*, the expected number of heterozygotes in the next generation t+1 is approximately

(Eqn 9) $$\begin{array}{l} \underset{\text{surviving heterozygotes}}{\underbrace{2\mathrm{Nx}\left(1-\theta \right)}}+\underset{\text{free spots}}{\underbrace{\mathrm{N\theta}}}\cdot \underset{\text{prob. that one parent is a heterozygote}}{\underbrace{\frac{2\cdot 2{\mathrm{xW}}_{m,t}}{2{\mathrm{xW}}_{m,t}+\left(1-2x\right)W_{r,t}}}}\\ \;\cdot \underset{\text{prob. that mutant allele is passed on}}{\underbrace{\frac{1}{2}}} \end{array}$$

(Eqn 10) $$\approx \;2\mathrm{xN}\cdot \underset{≕\lambda_{t}}{\underbrace{\left(1-\theta +\theta \frac{W_{m,t}}{W_{r,t}}\right)}}$$

where $W_{m,t}$ and $W_{r,t}$ are the fitness of the mutant‐resident heterozygote and of the resident homozygote at time *t*, as given by (7), and where we have assumed that *x* is close to zero such that 1−x≈1 and the term in the denominator is approximately $W_{r,t}$. Thus, the number of heterozygotes (and equivalently the frequency of the rare allele) grows by a factor $\lambda_{t}$.

To check whether the mutant can invade from low frequency, we then need to compute the geometric mean $\tilde{\lambda }$ of $\lambda_{t}$ over time. In the case of a single herbivore with occurrence probability $p_{1}$, the mutant can invade if

(Eqn 11) $$\begin{array}{rcl} \tilde{\lambda } & = & {\underset{\text{growth factor if herbivore is present}}{\underbrace{\left(1-\theta +\theta \cdot \frac{b\left(a_{1,m}\right)f\left(z_{c,m}\right)}{b\left(a_{1,r}\right)f\left(z_{c,r}\right)}\right)}}}^{p_{1}}\\ & & \cdot {\underset{\text{growth factor if herbivore is absent}}{\underbrace{\left(1-\theta +\theta \cdot \frac{f\left(z_{c,m}\right)}{f\left(z_{c,r}\right)}\right)}}}^{1-p_{1}}>1, \end{array}$$

where the activity and cost traits are computed using (1), (2), (5), and (6). If every homozygous resident type can be invaded by at least one mutant that differs from the resident at one locus, we score polymorphism as possible. That is, no multi‐locus genotype can go to fixation in the population and we predict genetic polymorphism at at least one of the loci. One could also do an extended invasion analysis with mutants differing at multiple loci, but such mutants are unlikely to arise for small mutation rates.

With nonoverlapping generations $\left(\theta =1\right)$ and the same effect size $m_{l1}=1$ for all loci, (11) can be further simplified and there is scope for polymorphism if

(Eqn 12) $$b{\left(r+d_{a}\right)}^{p_{1}}\cdot f\left(r+d_{c}\right)>b{\left(r\right)}^{p_{1}}\cdot f\left(r\right)$$

or

(Eqn 13) $$b{\left(r-1+d_{a}\right)}^{p_{1}}\cdot f\left(r-1+d_{c}\right)>b{\left(r\right)}^{p_{1}}\cdot f\left(r\right),$$

where r is the number of 11 loci in the resident. If r is 0 or *L*, only one of the possible mutants exists and so only one of the conditions needs to be checked.

### Individual‐based simulations

The analytical results only tell us under which conditions the population would be expected to be fully monomorphic or somewhat polymorphic. But it cannot tell us at how many loci polymorphism will arise and how patterns of allele frequencies will look like in the face of random genetic drift. Also, the analytical approach does not work with different effect sizes of the different loci against the different herbivores. To investigate these aspects, we built a stochastic individual‐based simulation in C++. For each parameter combination, we ran five replicate simulations for 5000 generations with a population size N=500, g=5 generations per phase, a mutation rate of 0.0001, and 10 unlinked loci. Like many population genetic simulations, we work with a relatively small population size and a relatively large mutation rate to keep simulation run times down. Since evolutionary processes often only depend on the product of population size and mutation rates, these settings can approximate larger natural populations with a lower mutation rate (Johnson *et al*., 2024).

For each parameter combination, we then quantified or estimated (1) the total number of ‘metabolites’, that is how many of the 10 loci have some 1 alleles in the population (γ diversity), (2) the average number of ‘metabolites’ per individual (also between 0 and 10), estimated as $\sum_{l=1}^{L}1-{\left(1-\pi_{l}\right)}^{2}$, where $\pi_{l}$ is the frequency of the 1 allele at locus *l* (α diversity), and (3) the average number of ‘metabolites’ that are not shared in a randomly drawn pair of individuals (i.e. one individual has it, the other one does not), estimated as $\sum_{l=1}^{L}2{\left(1-\pi_{l}\right)}^{2}\cdot \left(1-{\left(1-\pi_{l}\right)}^{2}\right)$ (β diversity). We evaluated each measure at the end of the simulation run and averaged across the five replicates. To generate null expectations for the diversity measures, we ran 500 replicates of a neutral model with all protective effects $m_{\mathrm{li}}$ as well as the cost parameter *c* set to zero. We also ran simulations where dominance coefficients were not the same across loci but independently drawn for each locus and independently for activity and costs from a uniform distribution on the interval between 0 and 1.

## Results

### Testing the fluctuating selection and dominance reversal hypotheses

**Fig. 2:**
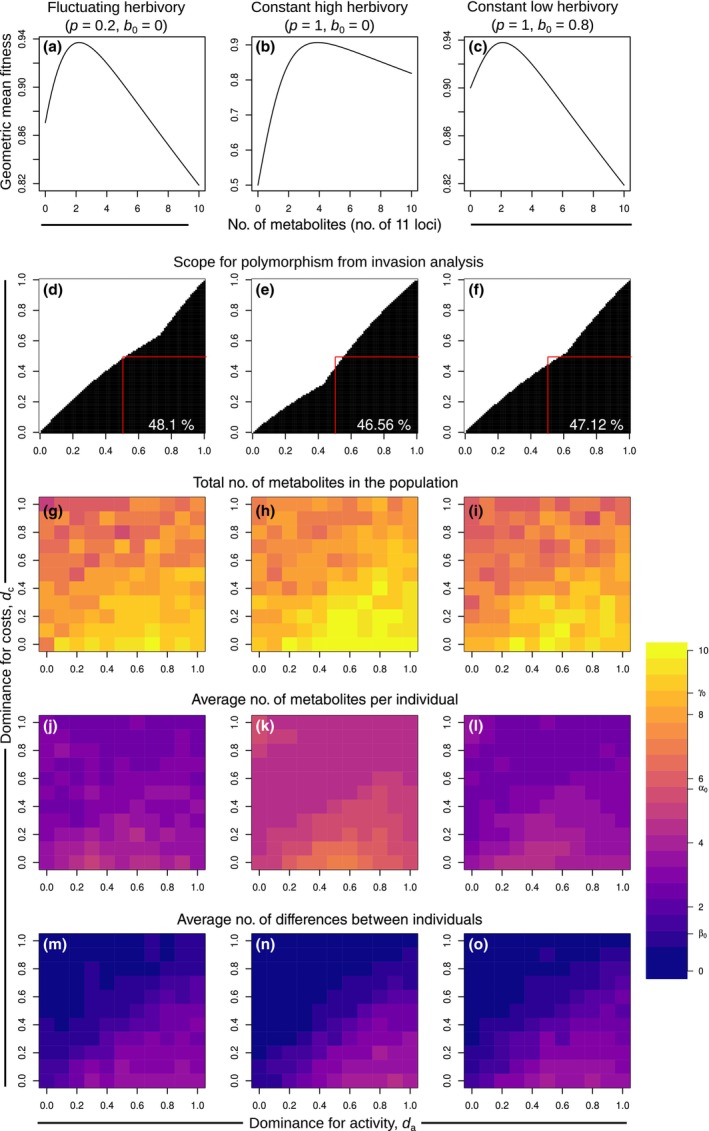
Dependence of chemodiversity patterns on dominance coefficients and fluctuations in the presence of one herbivore. *b*_0_ represents the baseline probability to escape the herbivore and *p* represents the probability that the herbivore is present and active during a particular phase. (a–c) Geometric mean fitness of homozygotes with different numbers of protection alleles. (d–f) Combinations of dominance coefficients for which the analytics predict that polymorphism is possible are shown in black. The proportion of dominance combinations for which polymorphism is possible is indicated as a percentage in the bottom right. The red square indicates the parameter space where polymorphism would be expected under the reversal of dominance hypothesis. (g–o) Results of the corresponding stochastic individual‐based simulations. The average values under neutrality of the total number of metabolites in the population, the average number of metabolites per individual, and the average number of differences between individual are indicated as γ_0_, α_0_, and β_0_ on the color scale.

We first focus on the simplest case of a single herbivore $\left(n_{h}=1\right)$ with every locus having the same antiherbivore effect $m_{l,1}=1$ and address the two population genetic hypotheses, that is the fluctuating selection hypothesis and the dominance reversal hypothesis (Fig. 2). To test for the effect of fluctuations, we compared three scenarios. In the fluctuating herbivory scenario (left column of Fig. 2), the herbivore was present in 20% of phases and the baseline probability of escaping the herbivore when it is present was low ($b_{0}=0$, blue curve in Fig. 1a). In the constant high herbivory scenario (middle column), we used the same benefit function, but the herbivore was present in every phase, that is there were no fluctuations. In the constant low herbivory scenario (right column), the herbivore was also present in all phases, but the baseline probability to escape the herbivore was higher (green curve in Fig. 1a) such that the overall herbivory pressure was similar to the fluctuating herbivory scenario. As might be expected, in the fluctuating herbivory scenario and in the constant low herbivory scenario, the optimum number of metabolites based on geometric mean fitness was similar and lower than in the constant high herbivory scenario (upper row of Fig. 2).

To test the dominance reversal hypothesis, we varied the dominance coefficients for benefits and costs independently (second row of Fig. 2). According to the analytic approach, polymorphism requires the dominance coefficient for activity $d_{a}$ to be roughly as high or higher than the dominance coefficient for costs $d_{c}$ (black region). Based on the dominance reversal hypothesis, one would have expected that polymorphism is only possible when dominance for activity is above 0.5 and dominance for costs is below 0.5 (red squares). However, the actual coexistence region was larger, while also excluding some parameter combinations with $d_{a}>0.5$ and $d_{c}<0.5$. Against the expectation from the fluctuating selection hypothesis, the region where polymorphism is possible was only slightly higher in the fluctuating herbivory scenario compared with the two constant herbivory scenarios. A sufficient difference between dominance for activity and dominance for costs could maintain polymorphism even in a constant environment.

The corresponding individual‐based simulation results (bottom three rows of Fig. 2) are qualitatively consistent with the analytical results. Again, there was little difference between the fluctuating herbivory and constant low herbivory scenario. This result also held up with overlapping generations and under a generalist‐specialist trade‐off (Supporting Information Notes S1 with Fig. S1 and Table S1 and Notes S2 with Figs S2, S3). The total number of metabolites in the population (third row) and the average number of metabolites per individual (fourth row) were highest in the constant high herbivory scenario. The average number of metabolites not shared between individuals (bottom row) was close to zero in regions where the analytical results predict that polymorphism is not possible, but reached values of four or more in regions where polymorphism is predicted. Roughly in this region, the average number of metabolites in the population and the average number of differences between individuals were also higher than expected under neutrality. The average number of metabolites per individual exceeded neutral expectations only in the constant high herbivory scenario. In regions not conducive to polymorphism, all diversity levels were generally below neutral expectations.

### Testing the interaction diversity hypothesis

Next, to test the interaction diversity hypothesis, we ran simulations with one, two, or five herbivores. In addition to the three fluctuation (or not) scenarios, we considered two scenarios for the effect size distributions: In the ‘only repellent’ scenario, each locus had protective effects 0.2, 0.4, 0.6, 0.8, or 1 against each herbivore with probability 0.1 each, and was neutral with probability 0.5. In the ‘repellent + attractive’ scenario, the probabilities for the different protective effect sizes were the same, but each metabolite also had attractive effects of magnitude 0.2, 0.4, 0.6, 0.8, and 1 with probability 0.02 each, and the probability to be neutral was 0.4 (see Fig. S4 for a visualization of the two distributions). The values were independently drawn from these distributions for each combination of locus, herbivore, and replicate. That is, a single metabolite could have different effects on the different herbivores.

The results were qualitatively similar to those in Fig. 2 here and in other scenarios below, with the difference between dominance for activity and dominance for costs as main driver for polymorphism. To better understand the effect of the number of herbivores, we summarized all the results by averaging the three quantities of interest (total metabolites, average number per individual, and average number of differences between individuals) across all dominance parameter combinations. We also obtained SE of the mean based on average chemodiversity measures across dominance combinations for each of the five replicates. Because of the many independent simulations going into each replicate, SE are very small. In addition, we obtained the scope of polymorphism analytically as the % of dominance parameter space for which the analytical calculations predict polymorphism (*c*. numbers in Fig. 2d–f).

**Fig. 3:**
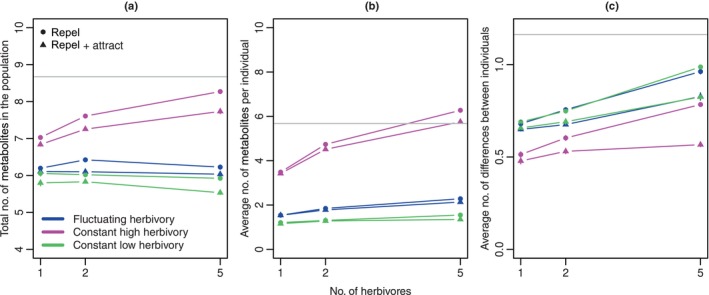
Effect of the number of herbivores and the distribution of effect sizes on chemodiversity patterns. (a) Total number of metabolites in the population; (b) average number of metabolites per individual; (c) average number of differences between individuals. The bars indicate the SE, but many of them are so small that they fall entirely inside the point and are therefore not visible. Note that the points are connected for increased visual clarity, but that nonlinear and even nonmonotonic relationships are possible. The gray horizontal lines indicate average diversity metrics under a neutral model.

As predicted under the interaction diversity hypothesis, the average number of metabolites per individual increased with the number of herbivores, albeit only very weakly under constant low herbivory (Fig. 3b). The interaction diversity hypothesis does not make a clear prediction for differences between individuals, but our model indicates that the average number of differences between individuals increased with the number of herbivores under all scenarios. The effect of the number of herbivores on the total number of metabolites in the population differed among scenarios: More herbivores led to more metabolites under constant high herbivory, but had little effect under fluctuating herbivory. Unexpectedly, an increasing number of herbivores slightly decreased metabolite numbers under constant low herbivory in the repel + attract scenario. With increasing herbivore numbers, the probability for a metabolite to attract at least one herbivore increases (0.1 for 1 herbivore, 0.19 for 2 herbivores, and 0.41 for 5 herbivores), while the probability that such loci are lost stays roughly constant (0.49, 0.48, 0.52). Thus, with more herbivores, there are fewer metabolites that are unconditionally beneficial. This can lead to a higher loss probability of presence alleles and therefore lower total numbers of metabolites in the population.

Diversity levels were again in most cases lower than under neutrality. Since these diversity levels are averages over the entire dominance parameter space, it seems that the diversity increases in the region conducive to polymorphism are often outweighed by the diversity decreases in the rest of parameter space (Fig. 2).

For the case of a single herbivore, we also explored the relationship between antiherbivore effect of a metabolite and final allele frequency of the corresponding presence allele (Fig. S5). While metabolites with an attractive or small protective effect were restricted to low allele frequencies, metabolites with increasing protective effect tended to have higher final allele frequencies.

The results were similar when dominance coefficients were not constant across loci but drawn independently from a uniform distribution (Fig. S6), presumably because still roughly the same overall proportion of loci had parameters conducive to polymorphism. In the fluctuating herbivory scenario with only repellent effects (the other scenarios yield very similar results), for most loci that are polymorphic (which we define here as having allele frequencies between 0.1 and 0.9), dominance for activity was larger than dominance for costs (Fig. S7).

### Testing the synergy hypothesis

**Fig. 4:**
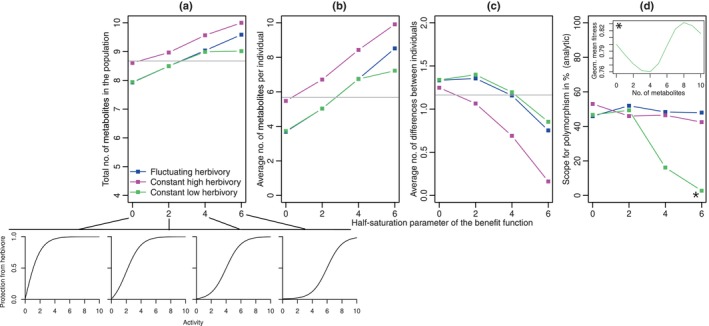
Effect of the shape of the benefit function on patterns of chemodiversity in the case of a single herbivore. a) Total number of metabolites in the population; (b) Average number of metabolites per individual; (c) Average number of differences between individuals; (d) Scope for polymorphism according to the analytic invasion analysis. With increasing half‐saturation parameter $a_{\text{half}}$, the benefit function becomes more synergistic (see small plots below a). Because the slope of the logistic function is steepest around the half‐saturation function, a smaller half‐saturation coefficient leads to higher protection at lower total activity. The bars indicate the SE, but many of them are so small that they fall entirely inside the point and are therefore not visible. The inset in (d) shows geometric mean fitness for the various homozygote genotypes with different number of metabolites for the constant low herbivory scenario with $a_{\text{half}}=6$ (see *). Note that the points are connected for increased visual clarity, but that nonlinear and even nonmonotonic relationships are possible. The gray horizontal lines indicate average diversity metrics under a neutral model.

To explore patterns of chemodiversity under the synergy hypothesis, we focused on a single herbivore and compared different shapes of the benefit function (small plots below Fig. 4). Scenarios with a low half‐saturation constant $a_{\text{half}}$ had diminishing returns of antiherbivore protection with increasing number of metabolites. With high half‐saturation constant, the benefit of having multiple metabolites was larger than the benefit expected from single metabolite effects over most of the range (synergistic effects). Had we chosen $b_{0}=0$, like in most other simulations, the scenarios would have differed not only in the shape but more strongly also in the overall protection level against herbivores. Thus, we chose $b_{0}$ for each scenario such that individuals with activity 0 always had probability 0.01 to escape the herbivore when it is present.

Both under constant or fluctuating herbivory, the total number of metabolites in the population and the average number of metabolites per individual increased with the half‐saturation parameter of the benefit function, that is as the benefit function becomes more synergistic, and also exceeded neutral levels for high half‐saturation parameters. By contrast, the average number of differences between individuals was above the neutral level for low half‐saturation constants but decreased as the benefit function becomes more synergistic. Fluctuating herbivory and constant low herbivory produced similar chemodiversity patterns, but for strongly synergistic benefit functions, the total number and average number per individual were higher under fluctuating herbivory. Constant high herbivory led to the highest number of metabolites, both in total and per individual, but the fewest differences between individuals. The analytic approximation (Fig. 4d) did not capture the between‐individual chemodiversity well and predicted that the scope for polymorphism is lowest in the constant low herbivory scenario. For the synergistic benefit function, the geometric mean fitness function can be nonmonotonic (see inset), suggesting that the extreme genotype without any metabolites might be noninvasible by all one‐step mutants, but could be invasible by mutants differing in multiple positions. In the simulations, the mutation rate seems to be large enough that multiple mutants differing in several positions segregate in the population. Apparently, other genotypes invaded the no‐metabolite genotype in many cases where the analytical results suggest it is not possible. Again, the results were similar to those with dominance coefficients drawn from a uniform distribution (Fig. S8) and most of the loci with allele frequencies between 0.1 and 0.9 had a larger dominance coefficient for activity than for costs (Fig. S9).

### Testing the screening hypothesis

A more complex model with metabolic pathways is needed to test most predictions of the screening hypothesis. However, we can test one prediction: Based on the screening hypothesis, plants with more metabolites should have a higher probability of having a metabolite that is active against a newly colonizing herbivore and therefore a higher fitness after such an invasion (Jones & Firn, 1991). To test this, we ran simulations under the fluctuating herbivory scenario and with varying dominance parameters, where 1, 2, or 5 herbivores were present until time 5000 and an additional herbivore was introduced at time 5000 and present until the end of the simulation at time 5500. For these simulations, we used the distributions of effect sizes from Fig. S4 and the effect of each metabolite on each herbivore was drawn independently, also for the additional herbivore.

**Fig. 5:**
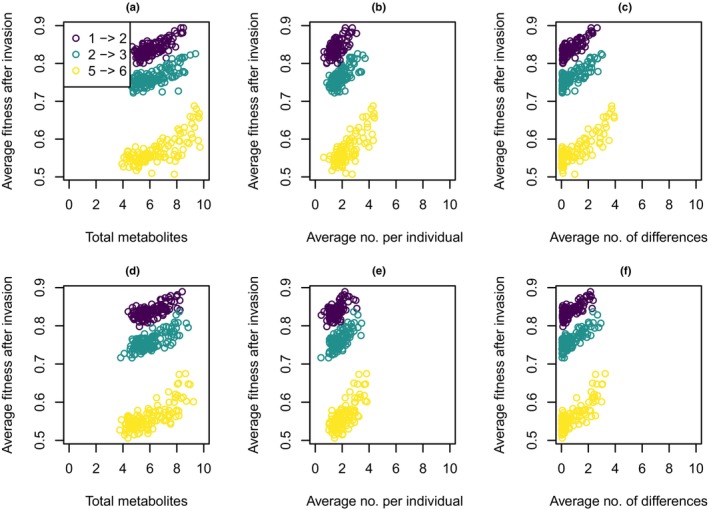
Average fitness over 500 generations after the invasion of a new herbivore as a function of the different aspects of chemodiversity before invasion (average over the last 95 generations before invasion). Each point corresponds to the average over five replicates for one of the dominance and herbivore number scenarios. The three colors correspond to different numbers of herbivores before and after the invasion (see legend). (a–c) Scenario with only repellent effects of metabolites on herbivores as depicted in Fig. S4(a). (d–f) Scenario with both repellent and attractive effects of metabolites on herbivores as depicted in Fig. S4(b).

As predicted, plant populations with a higher chemodiversity (independently of whether it was measured as total metabolites produced, average number of metabolites produced per individual, or average number of differences between individuals) maintained higher average fitness in the time period after the invasion of an additional herbivore (Fig. 5). This was the case both when metabolites had only repellent effects (top row of Fig. 5) and when metabolites had both repellent and attractive effects (bottom row of Fig. 5).

## Discussion

### Support for and new predictions of the five hypotheses

We have presented a stochastic population genetics modeling approach to qualitatively test various verbal models and hypotheses on the evolution of chemodiversity.

Our analytical and simulation results agree with the dominance reversal hypothesis in that differences in dominance between different traits, here the costs and benefits of defense metabolites, are a key factor for the maintenance of polymorphism. However, the exact conditions did not match with the hypothesis. We had expected polymorphism when dominance for costs is below 0.5 and dominance for activity above 0.5. But polymorphism actually emerged when the dominance for antiherbivore activity was roughly as large or larger than the genetic dominance for the costs of producing metabolites, that is heterozygotes experienced relatively large benefits and relatively low costs of their one copy of the metabolite‐producing allele. This led to a high total number of metabolites produced in the population and a high average number of differences between individuals. Beneficial reversal of dominance was not necessary, but also not completely sufficient for the maintenance of polymorphism.

Antagonistic pleiotropy with changes in dominance between traits is known to be a powerful mechanism for the maintenance of genetic polymorphism (Rose, 1982; Curtsinger *et al*., 1994). Although the focus has been mostly on reversals of dominance, a closer examination of the model by Rose (1982) shows that also in a simpler model with antagonistic pleiotropy at a single locus in a constant environment, smaller quantitative differences in dominance between traits can be sufficient to maintain polymorphism (Notes S3 with Table S2 and Fig. S10). Similar observations were made by Van Dooren (2006) and Brud (2023). Thus, one should talk less about beneficial dominance reversals and more about beneficial dominance shifts as a factor promoting genetic variation. Until recently, the maintenance of polymorphism by antagonistic pleiotropy with dominance reversals has been seen more as a strange exception rather than as an important mechanism contributing to diversity levels in natural populations (Curtsinger *et al*., 1994; Hedrick, 1999). However, there are more and more empirical examples of dominance reversal across contexts and theoretical models showing how it can easily arise (see recent reviews by Connallon & Chenoweth, 2019; Grieshop *et al*., 2024).

Differences in dominance that according to our model are often sufficient for maintenance of polymorphism, can arise even more easily than a reversal of dominance. Such differences in dominance have been observed for example for loci influencing mandibular morphology in mice (Ehrich *et al*., 2003) and can arise in branched enzyme pathways with nonlinearities and feedbacks (Keightley & Kacser, 1987). In fact, since many traits and fitness components emerge from highly nonlinear processes, there is no particular reason why dominance should be equal for all traits affected by a pleiotropic locus. For metabolic pathways in particular, heterozygotes might be expected to be intermediate with respect to the costs of genes and enzymes (corresponding to a dominance for costs of 0.5 in our model). Flux through the respective pathway is often a concave (diminishing returns) function of enzyme concentration (Kacser & Burns, 1981). Thus heterozygotes will be closer in flux to the homozygote with the larger flux (in our model the presence‐presence homozygotes) and dominance for activity is expected to be larger than 0.5 (Kacser & Burns, 1981). This is the case as long as the enzyme is not saturated with substrate (Wright, 1934; Kacser & Burns, 1981; Cornish‐Bowden, 1987). In specialized metabolism, it is indeed expected that most enzymes operate far from saturation. First, flux through a specialized metabolite pathway is frequently controlled at gateway entry points (Olson‐Manning *et al*., 2013; Dixon & Dickinson, 2024). Second, compared with enzymes in primary metabolism, enzymes in specialized metabolism have on average less optimized kinetic parameters, suggesting that selection pressure on efficiency in specialized metabolism is relatively low, presumably because of the lower flow through these pathways (Bar‐Even *et al*., 2011; Bar‐Even & Salah Tawfik, 2013). Thus, it appears plausible that enzymes have not evolved Michaelis constants $K_{M}$ low enough to match the physiological substrate concentrations and thus do not operate at saturation, again making dominance for activity likely.

Unfortunately, there are to our knowledge only few studies so far that quantify the phenotypes or fitness of heterozygotes relative to homozygotes at loci underlying plant secondary metabolite variation. Kondra & Stefansson (1970) crossed two cultivars of *Brassica napus*, one with very low and one with relatively high concentrations of three glucosinolates. Offspring glucosinolate content was more than the average between the two parents and the results were consistent with an inheritance model with recessive absence alleles. Similarly, heterozygotes for loss‐of‐function mutations in the aliphatic glucosinolate pathway of *Arabidopsis thaliana* produced substantially more than half of wild‐type levels for most metabolites although gene expression was often close to half or even less, which would be consistent with dominance for costs at or below 0.5 and dominance for antiherbivore activity above 0.5 (Olson‐Manning *et al*., 2013). More such studies are urgently needed to make progress in understanding the evolution of chemodiversity, but so far both theoretical enzyme kinetic considerations and the available empirical data suggest that changes in dominance conducive to high chemodiversity and genetic polymorphism frequently occur in nature. To our knowledge, this is the first time that dominance changes are highlighted as a potential key factor in the evolution of plant chemodiversity.

Contrary to the expectation from the fluctuating selection hypothesis, temporal fluctuations in herbivore presence had surprisingly small effects for chemodiversity in our model. The reason seems to be that selection pressures under fluctuating herbivore occurrence are in the long run similar to scenarios with continuous presence of herbivores but lower herbivory pressure (Figs 2, 3). Had previous studies on the interplay of temporally fluctuating selection and dominance changes (Hedrick, 1976; Wittmann *et al*., 2017) also included such a constant control treatment, they might have found similar maintenance of polymorphism even without fluctuations. However with multiple herbivores or strong synergy, fluctuating herbivore occurrence slightly promoted chemodiversity compared with constant low herbivory both within individuals and within populations, but not between individuals (Figs 3, 4). While we have focused on a scenario where herbivore presence fluctuates on a longer time scale than plant generations (g=5 plant generation before a random shift in herbivore absence or presence), the fact that diversity levels under fluctuating herbivore were very similar to constant low herbivory and that our analytic condition for polymorphism was entirely independent of *g* suggests that long‐term chemodiversity patterns are robust to the details of the fluctuation regime. In a model with antagonistic pleiotropy driven by a fecundity–viability trade‐off of flowering time, Brown & Kelly (2018) similarly found only a small effect of environmental fluctuations on the maintenance of polymorphism. Since many studies on plant chemodiversity speculate about an important contribution of fluctuating selection to maintaining chemodiversity, it is important to recognize that the conditions for this to happen might be rather limited.

As expected from the interaction diversity hypothesis (Whitehead *et al*., 2021), with increasing number of herbivores, the average number of metabolites per individual increased (Fig. 3). Surprisingly, this did not always lead to a higher total number of metabolites in the population, apparently because some of the scenarios with more herbivores also caused a larger probability of loss of rare alleles. Maybe the most interesting result of the simulations with multiple herbivores is that increasing herbivore numbers do not just promote an increase in the number of metabolites per individual, a straightforward prediction, but also lead to more differences in metabolite composition between individuals, a nontrivial prediction that would not be possible without mathematical models such as ours.

Consistent with the synergy hypothesis, our model produced generally larger numbers of metabolites in the population and per individual when metabolites had synergistic effects on protection against herbivores (Fig. 4). However, the average number of differences between individuals decreased with increasing synergy. This is also expected since when most individuals have most metabolites, they cannot differ in many metabolites.

Lastly, our simulation results support one of the predictions of the screening hypothesis, namely that more chemodiverse plant populations cope better with the invasion of an additional herbivore with randomly drawn traits. However, this benefit of chemodiversity is not the reason for the evolution of chemodiversity in our model. Chemodiversity evolved in response to the ‘native’ herbivores and if, because of the parameter settings, a higher diversity evolved, this coincidentally also helped to cope with a new herbivore, which is rather self‐evident. To tackle the core of the screening hypothesis, more sophisticated models also involving the structure of metabolic pathways are needed.

### Limitations and outlook

Here, we have taken a simplified approach where the genotype directly determines whether or not a metabolite is produced. In reality, the link between genotype and chemotype may not be so direct. For example, if metabolites are produced by multi‐step enzymatic pathways, a metabolite can only be produced if the enzyme catalyzing its synthesis from its direct precursor is expressed, but also all precursors need to be present and the enzymes necessary to produce them need to be expressed. Thus, metabolites produced in complex pathways do not evolve independently. A logical next step is to develop more mechanistic models that do not take the shortcut from genotype to metabolite profile, but model the underlying proteome and metabolic pathways. Such models could then be used to test other predictions of the screening hypothesis and to model quantitative variation in addition to qualitative presence–absence variation. Such models should also allow for the evolution of gene regulation to test whether plant populations evolve to defend themselves with few highly expressed defenses or many weakly expressed ones. Our analysis of the relationship between metabolite effect strength and final allele frequency (Fig. S5) suggests that small‐effect defenses tend rare in the population, thereby contributing to the average number of metabolites in the population, but not so much to the other chemodiversity measures. Metabolites with intermediate effect tend to have also intermediate allele frequencies and thus contribute substantially to differences between individuals, while large effect metabolites tend to have the highest frequencies and thus contribute especially to the average number of metabolites per individual.

Second, we have not considered that plants may be affected by the chemical profile and repellent or attractive effects of their neighbors, either through associational resistance or associational susceptibility (Underwood *et al*., 2014). These phenomena can under some conditions maintain plant defense polymorphisms via negative frequency‐dependent selection (Sato, 2018). So far, models for associational effects (e.g. Sato *et al*., 2017) have assumed asexual reproduction where dominance does not have to be considered. Since both frequency dependence from ecological interactions and genetic dominance patterns can be important for the maintenance of chemodiversity, an interesting future direction is to build diploid sexual reproduction models with associational effects that allow us to explore their interaction.

Third, we have assumed that plant population sizes are constant and herbivore pressures are independent of evolution in the plant population. Since eco‐evolutionary feedbacks for example with specialist herbivores can be important for the evolution of plant chemodiversity (Agrawal *et al*., 2013), it would be interesting to include population dynamics of plants and herbivores as well as plant–herbivore coevolution in future models.

Finally, our model allowed us to generate qualitative insights into the effects of ecological and genetic parameters on chemodiversity patterns. Now, empirical quantification of key model parameters such as dominance coefficients and the shape of the cost and benefit functions is needed in order to make quantitative predictions for chemodiversity patterns in natural plant populations.

## Competing interests

None declared.

## Author contributions

MJW and AB designed the research. MJW developed, programmed, and analyzed the model, and wrote the manuscript, with input from AB.

## Supporting information


**Dataset S1** Zip folder containing simulation code and analysis scripts.

**Fig. S1** Dependence of chemodiversity patterns on dominance coefficients and fluctuations in the presence of one herbivore and with overlapping generations with θ=0.3.
**Fig. S2** Patterns of chemodiversity in a generalist‐specialist scenario and different fluctuation scenarios.
**Fig. S3** Allele‐frequency spectra for generalist‐specialist scenarios and different fluctuation scenarios.
**Fig. S4** Effect size distributions used for simulations with multiple herbivores.
**Fig. S5** Relationship between the protective effect of a metabolite and its final allele frequency.
**Fig. S6** Effect of the number of herbivores and the distribution of effect sizes on chemodiversity patterns when the two dominance coefficients are independently drawn from a uniform distribution on [0,1].
**Fig. S7** Combinations of dominance for activity and dominance for costs for loci that were polymorphic and those that were not for different numbers of herbivores.
**Fig. S8** Effect of the shape of the benefit function on patterns of chemodiversity in the case of a single herbivore when the two dominance coefficients are independently drawn from a uniform distribution on [0,1].
**Fig. S9** Combinations of dominance for activity and dominance for costs for loci that were polymorphic and those that were not for different half‐saturation constants.
**Fig. S10** Changes in dominance can maintain polymorphism at a single locus with antagonistic pleiotropy, even without reversal of dominance.
**Notes S1** Results for the case with overlapping generations.
**Notes S2** Simulations with a generalist‐specialist trade‐off.
**Notes S3** Conditions for maintenance of polymorphism at a single locus with antagonistic pleiotropy in a constant environment.
**Table S1** Average chemodiversity levels across all dominance parameter combinations for different herbivory scenarios and with different adult death probabilities θ.
**Table S2** Fitness effects in a simple single‐locus antagonistic pleiotropy model with two fitness components (*W*_1_ and *W*_2_) that act either additively or multiplicatively.
Please note: Wiley is not responsible for the content or functionality of any Supporting Information supplied by the authors. Any queries (other than missing material) should be directed to the *New Phytologist* Central Office.

## Data Availability

Simulation code and analysis scripts are provided in a zip folder in Dataset S1.
