# Supplementary material for: How does plant chemodiversity evolve? Testing five hypotheses in one population genetic model
Source: New Phytol. 2024 Sep 5;245(3):1302–14. doi: 10.1111/nph.20096 (PMC11711931; doi:10.1111/nph.20096)
Supplement: Supplementary file 2 — Fig. S1 Dependence of chemodiversity patterns on dominance coefficients and fluctuations in the presence of one herbivore and with overlapping generations with θ=0.3. Fig. S2 Patterns of chemodiversity in a generalist‐specialist scenario and different fluctuation scenarios. Fig. S3 Allele‐frequency spectra for generalist‐specialist scenarios and different fluctuation scenarios. Fig. S4 Effect size distributions used for simulations with multiple herbivores. Fig. S5 Relationship between the protective effect of a metabolite and its final allele frequency. Fig. S6 Effect of the number of herbivores and the distribution of effect sizes on chemodiversity patterns when the two dominance coefficients are independently drawn from a uniform distribution on [0,1]. Fig. S7 Combinations of dominance for activity and dominance for costs for loci that were polymorphic and those that were not for different numbers of herbivores. Fig. S8 Effect of the shape of the benefit function on patterns of chemodiversity in the case of a single herbivore when the two dominance coefficients are independently drawn from a uniform distribution on [0,1]. Fig. S9 Combinations of dominance for activity and dominance for costs for loci that were polymorphic and those that were not for different half‐saturation constants. Fig. S10 Changes in dominance can maintain polymorphism at a single locus with antagonistic pleiotropy, even without reversal of dominance. Notes S1 Results for the case with overlapping generations. Notes S2 Simulations with a generalist‐specialist trade‐off. Notes S3 Conditions for maintenance of polymorphism at a single locus with antagonistic pleiotropy in a constant environment. Table S1 Average chemodiversity levels across all dominance parameter combinations for different herbivory scenarios and with different adult death probabilities θ. Table S2 Fitness effects in a simple single‐locus antagonistic pleiotropy model with two fitness components (W 1 and W 2) that act either [file NPH-245-1302-s002.pdf]

New Phytologist Supporting Information

Article title: How does plant chemodiversity evolve?
Testing five hypotheses in one population genetic model

Authors: Meike J. Wittmann and Andrea Bräutigam

Article acceptance date: 20 July 2024

Notes S1 Results for the case with overlapping generations

Results analogous to Fig. 2 for the case with overlapping generations where plants survive to the next time step with probability 0.7 (adult death probability $\theta = 0.3$) are shown in Fig. S1. While the analytical results show no major differences, the simulation results indicate that with overlapping generations and adult mortality 0.3 per time step all three chemodiversity measures are slightly lower than with non-overlapping generations. However, if the adult mortality was further decreased ($\theta = 0.07$) so that plants have an even longer average life span, all measures of chemodiversity are increased (Table S1). In both overlap scenarios, there was no clear difference in chemodiversity measures between the fluctuating herbivory scenario and the constant low herbivory scenario.

Table S1 Average chemodiversity levels across all dominance parameter combinations for different herbivory scenarios and with different adult death probabilities θ. TMP = average total number of metabolites in the population, MPI = average number of metabolites per individual, DM = average number of differences between individuals.

Herbivory scenario	θ	TMP	MPI	DM
Fluctuating	1	7.59	2.91	1.31
Constant high	1	8.27	4.70	1.26
Constant low	1	7.50	2.87	1.35
Fluctuating	0.3	6.55	2.87	1.01
Constant high	0.3	7.41	4.59	1.01
Constant low	0.3	6.46	2.77	1.04
Fluctuating	0.07	7.74	3.64	2.02
Constant high	0.07	8.74	5.48	2.02
Constant low	0.07	7.72	3.56	2.00

Previous theory has shown that temporally fluctuating selection can more easily maintain

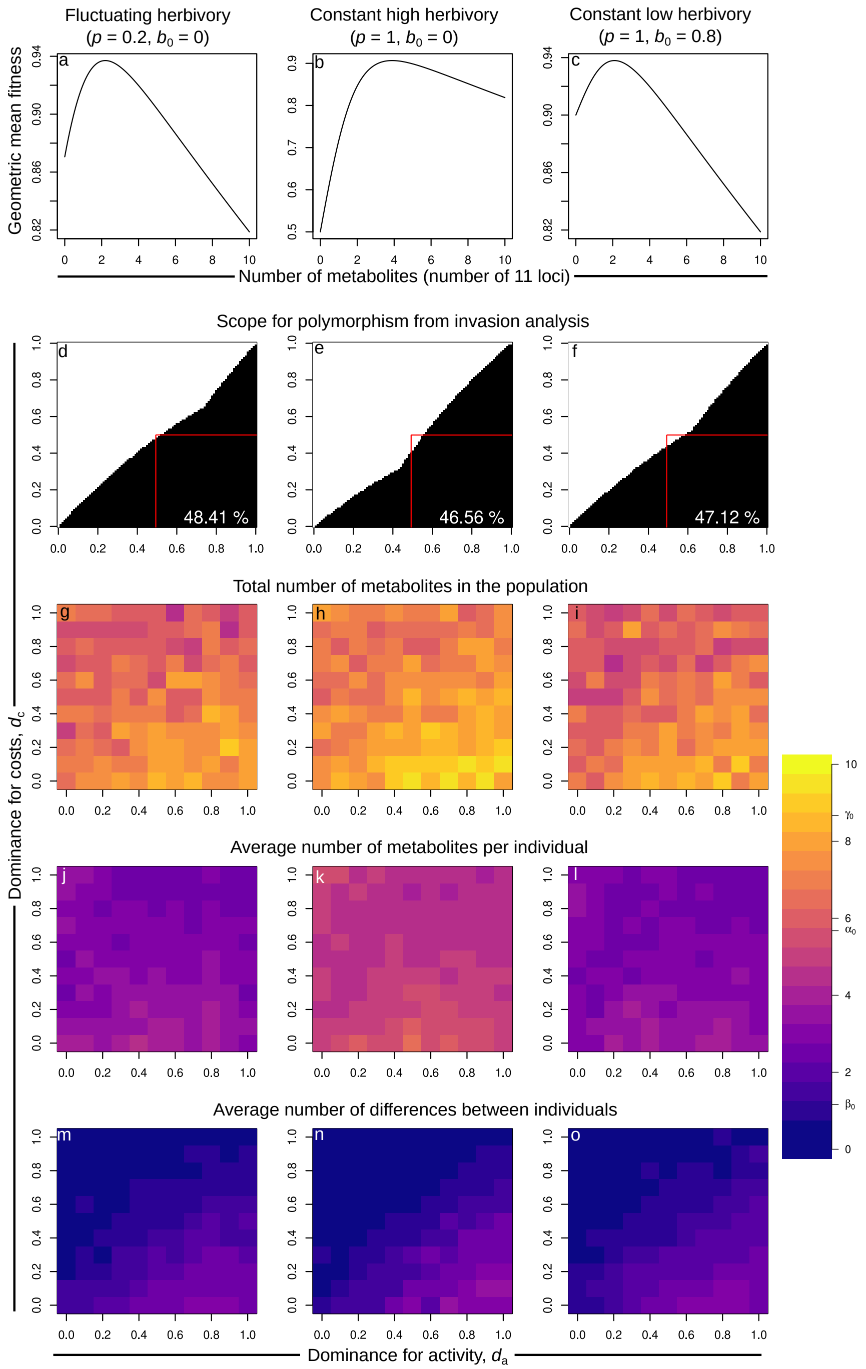

Figure S1 Dependence of chemodiversity patterns on dominance coefficients and fluctuations in the presence of one herbivore and with overlapping generations with $\theta = 0.3$. a-c: Geometric mean fitness of homozygotes with different numbers of protection alleles. d-f: combinations of dominance coefficients for which the analytics predict that polymorphism is possible. The proportion of dominance combinations for which polymorphism is possible is indicated as a percentage in the bottom right. g-o: Results of the corresponding stochastic individual-based simulations. The average values under neutrality of the total number of metabolites in the population, the average number of metabolites per individual and the average number of differences between individual are indicated as γ_0, α_0, and β_0 on the color scale.

genetic variation if generations are overlapping (Chesson and Warner 1981). Thus we had hypothesized that generation overlap would increase the parameter space where temporal fluctuations promote chemodiversity, but at least for the scenarios we investigated, this does not appear to be the case. For overlapping generations with a relatively large adult death probability, all chemodiversity measures were reduced while with a smaller adult death probability and thus more generation overlap, all chemodiversity measures were increased (Table S1). Thus, we would predict long-lived perennials to harbor more chemodiversity than annuals. However, long generation overlap increased chemodiversity in all herbivory scenarios with or without fluctuations. Thus, generation overlap did not increase the power of fluctuating selection to maintain chemodiversity. However, we have assumed here that herbivory only influences plant reproduction, but not the probability to survive to the next time step, which may not be realistic. More detailed modeling is needed to clarify how plant life history and fluctuating herbivory interact to shape chemodiversity.

Notes S2 Simulations with a generalist-specialist trade-off

To test whether a trade-off between generalist repulsion and specialist attraction, potentially with temporal fluctuations in specialist and generalist presence, promotes chemodiversity, we ran a scenario with two herbivores where all ten metabolites had the same effect sizes and affected both the generalist herbivore ($m_{l1} = 0.2$, 0.5, or 1) and the specialist herbivore ($m_{l2} = -0.2$).

For this, we generalised the analytical approach to the case of more than one herbivore:

$$\tilde{\lambda} = \prod_{\mathcal{H}} \left(1 - \theta + \theta \cdot \frac{\left(\prod_{i\in\mathcal{H}} b(a_{i,\mathrm{m}})\right) f(z_{c,\mathrm{m}})}{\left(\prod_{i\in\mathcal{H}} b(a_{i,\mathrm{r}})\right) f(z_{c,\mathrm{r}})}\right)^{\prod_{i\in\mathcal{H}} p_i \cdot \prod_{i\notin\mathcal{H}}(1-p_i)}, \tag{S1}$$

where the first product is over all possible subsets of herbivores $\mathcal{H}$. For example, with two herbivores

$$\tilde{\lambda} = \left(1 - \theta + \theta \cdot \frac{f(z_{c,\mathrm{m}})}{f(z_{c,\mathrm{r}})}\right)^{(1-p_1)(1-p_2)} \cdot \left(1 - \theta + \theta \cdot \frac{b(a_{1,\mathrm{m}}) f(z_{c,\mathrm{m}})}{b(a_{1,\mathrm{r}}) f(z_{c,\mathrm{r}})}\right)^{p_1(1-p_2)} \tag{S2}$$

$$\cdot \left(1 - \theta + \theta \cdot \frac{b(a_{2,\mathrm{m}}) f(z_{c,\mathrm{m}})}{b(a_{2,\mathrm{r}}) f(z_{c,\mathrm{r}})}\right)^{(1-p_1)p_2} \cdot \left(1 - \theta + \theta \cdot \frac{b(a_{1,\mathrm{m}}) b(a_{2,\mathrm{m}}) f(z_{c,\mathrm{m}})}{b(a_{1,\mathrm{r}}) b(a_{2,\mathrm{r}}) f(z_{c,\mathrm{r}})}\right)^{p_1 p_2}. \tag{S3}$$

When the repellent effect on generalists was only as strong as the attractive effect on specialists (0.2), individuals produced few metabolites and there were few differences between individuals in metabolites produced (Fig. S2 b, c), consistent with no scope for polymorphism according to the analytic calculations (Fig. S2 d). For stronger repellent effects on generalist herbivores, the number of metabolites produced in the population and per individual as well as the number of differences between individuals increased. For high protective effects against generalists, the constant high

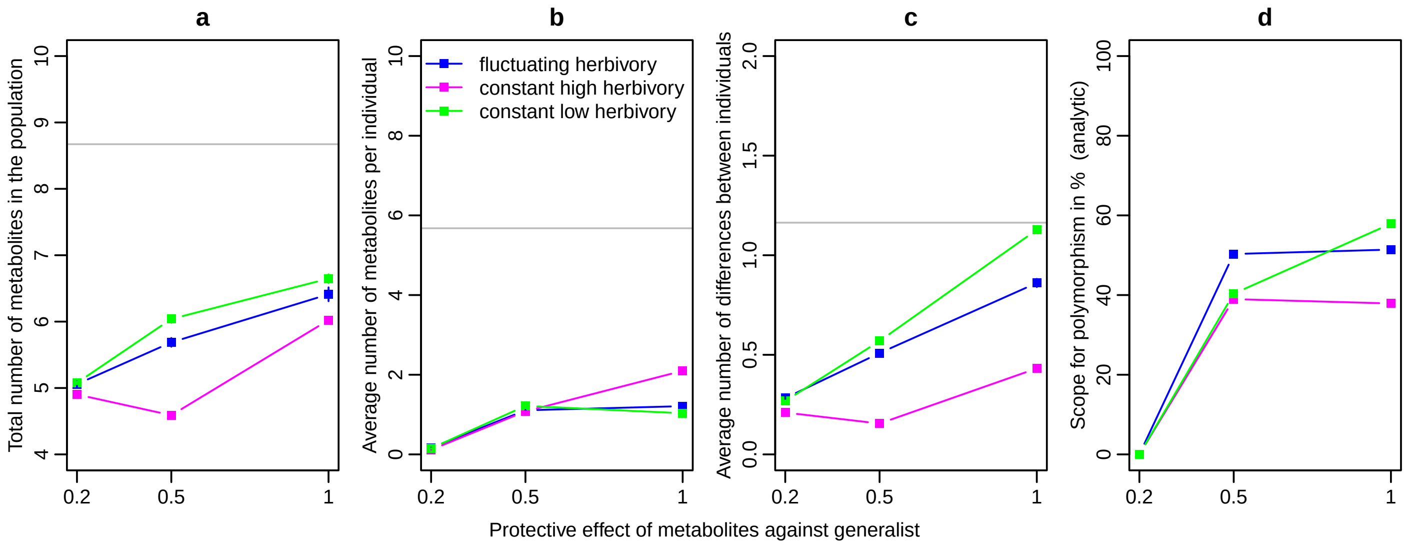

Figure S2 Patterns of chemodiversity in a scenario with one specialist which is attracted by metabolites with effect size -0.2 and one generalist. The magnitude of the protective effect against the generalist is varied on the x axis. The bars indicate the standard error, but many of them are so small that they fall entirely inside the point and are therefore not visible. Note that the points are connected for increased visual clarity, but that nonlinear and even nonmonotonic relationships are possible. The gray horizontal lines indicate average diversity metrics under a neutral model.

herbivory scenario had the highest number of metabolites per individual, but the fewest differences between individuals according to both simulations and analytical results. Again, there were no consistent differences between the fluctuating herbivory and constant low herbivory scenarios. The analytical results (Fig. S2 d) suggest that the generalist-specialist trade-off can slightly widen the dominance region where polymorphism is possible (compare to Fig. 2). Since the scope for polymorphism was in some cases above 50 %, there are some scenarios where polymorphism is possible although dominance for costs is slightly higher than dominance for activity.

Curiously, in the constant high herbivory scenario, the total number of metabolites in the population and the average number of differences between individuals was smallest at intermediate protective effect against generalists. A closer look at the underlying allele frequency distributions (Fig. S3 b) shows that in this scenario, there was usually one locus where the presence allele was fixed or at very high frequency, whereas most other loci were at 0 or very low frequency. For lower or higher protective effect against generalist herbivores, there were more loci with allele frequencies between 0.01 and 0.1 that thus contributed more to the total number of loci in the population and to individual differences. From these results, it is not clear why the allele frequency distribution in this case was so different, but it could be that in this scenario genotypes with two presence alleles at one locus are close to optimal, so that other genotypes are more strongly selected against

compared to other scenarios.

The average diversity levels were in all cases lower than under neutrality (gray lines).

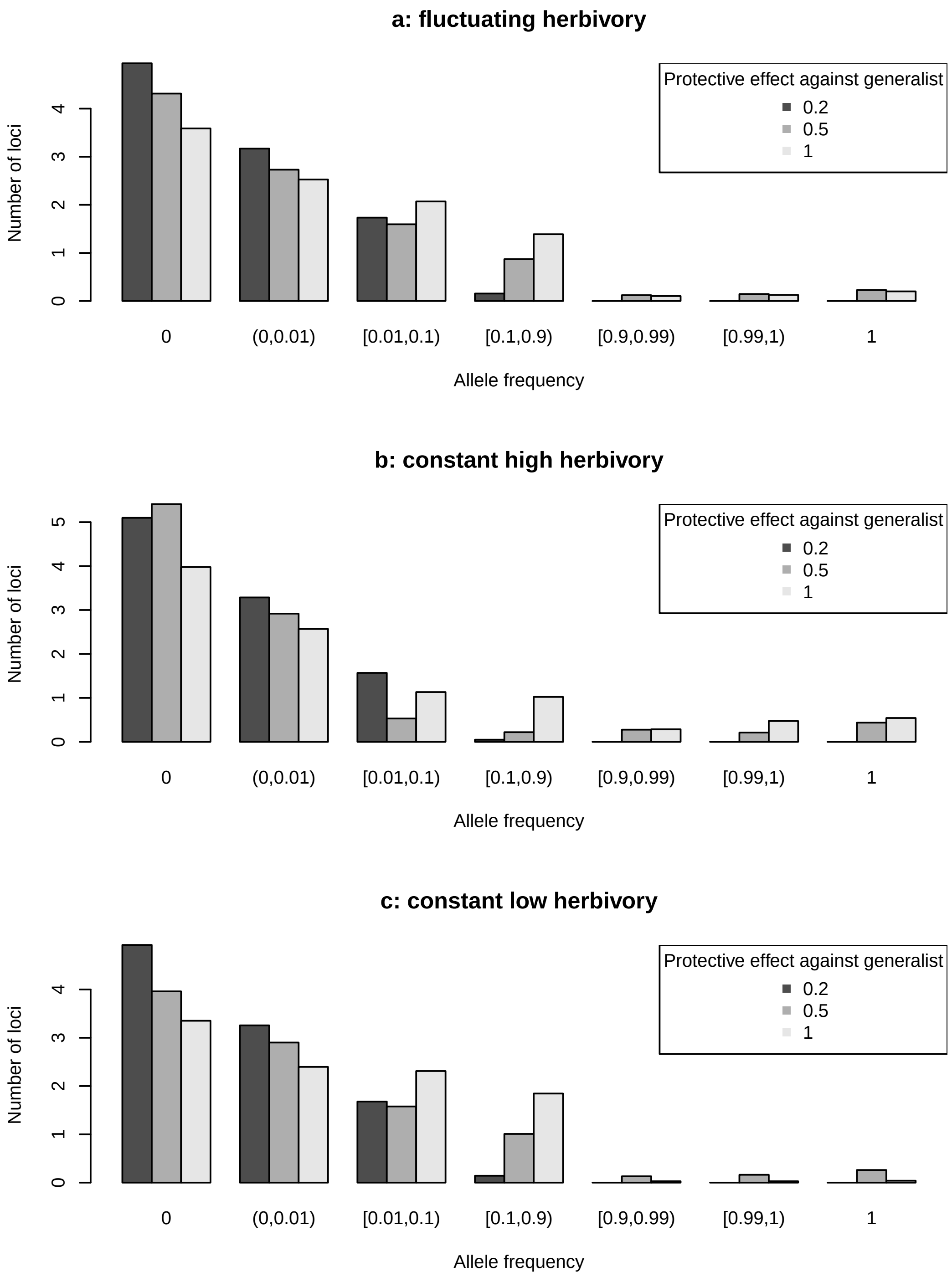

Figure S3 Allele-frequency spectra for generalist-specialist scenarios and different fluctuation scenarios. The underlying simulation results are the same as in Fig. S2. Each bar represents the number of loci (out of 10) that have allele frequencies in a given interval at the end of the simulation, averaged over all dominance parameter combinations and over the five replicates.

Additional simulation results for the interaction diversity hypothesis

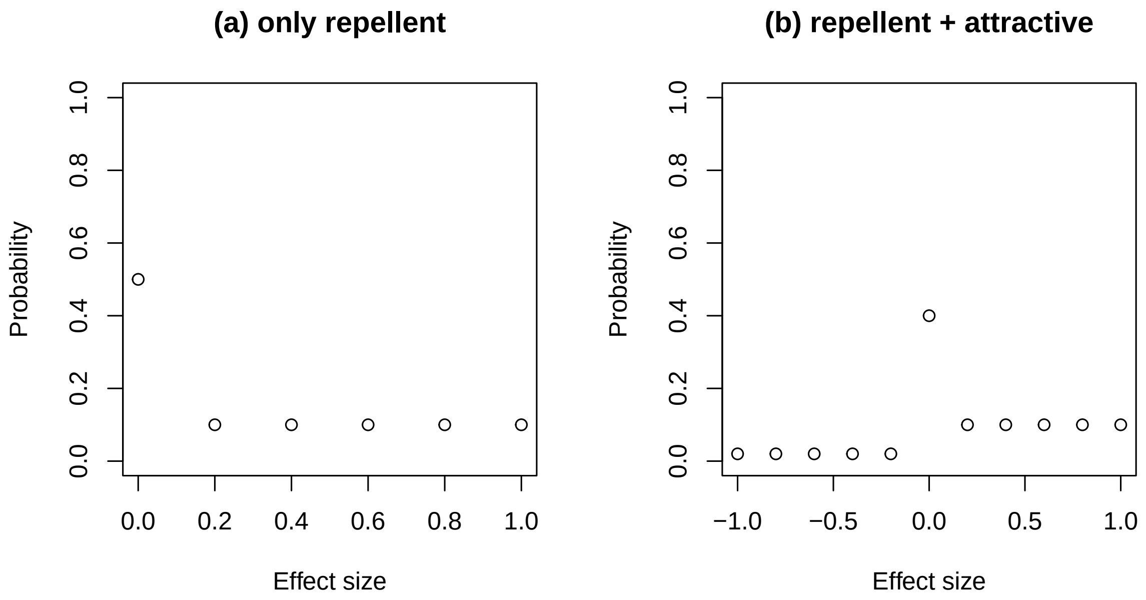

Figure S4 Effect size distributions used for simulations with multiple herbivores. Positive values correspond to protective effects against herbivores, whereas negative values correspond to attractive effects.

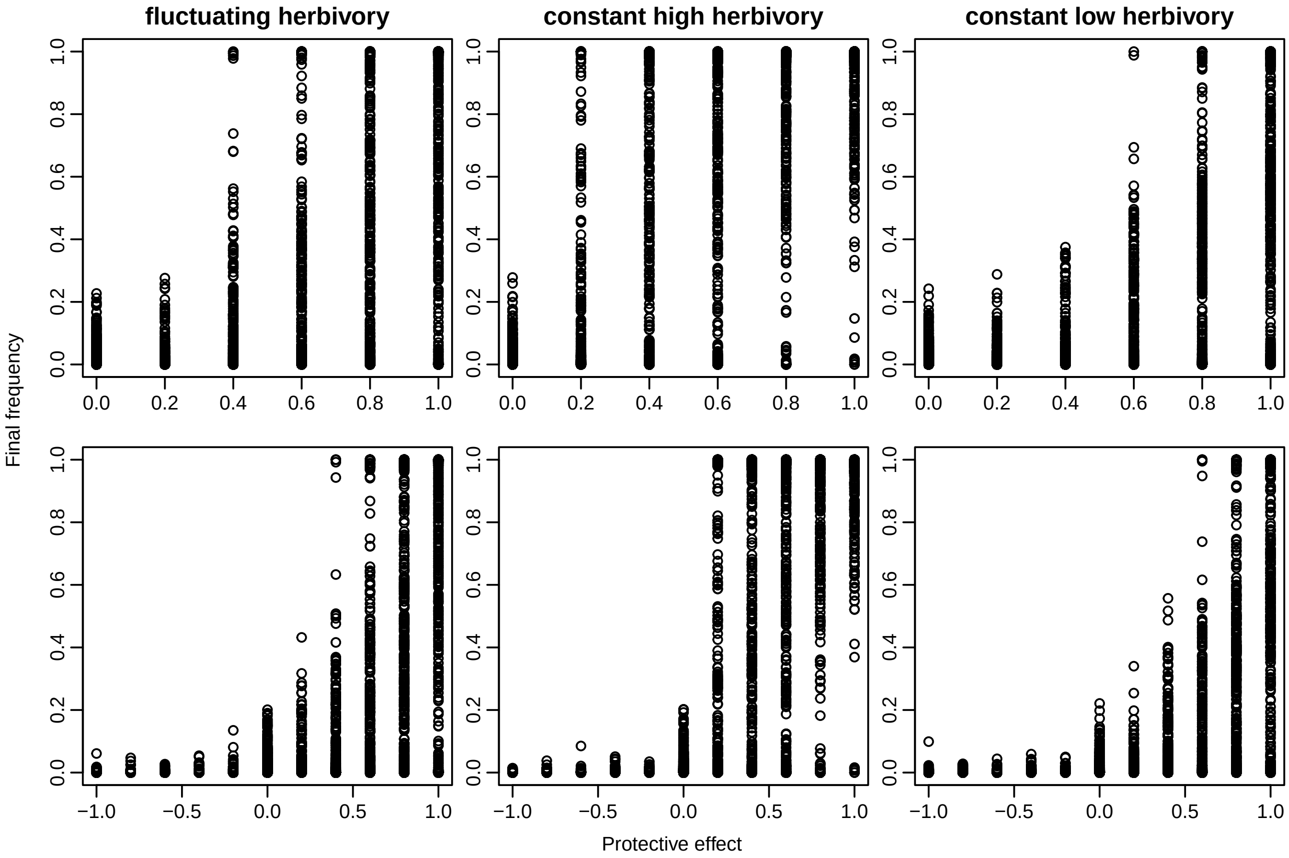

Figure S5 Relationship between protective effect of a metabolite and the final allele frequency of the presence allele in the case of a single herbivore when the two dominance coefficients are independently drawn from a uniform distribution on [0,1]. Each point corresponds to one locus in one of the replicates. Top row: Distribution with only repellent effects (see Fig. S4 a) Bottom row: Distribution with both repellent and attractive effects (see Fig. S4 b).

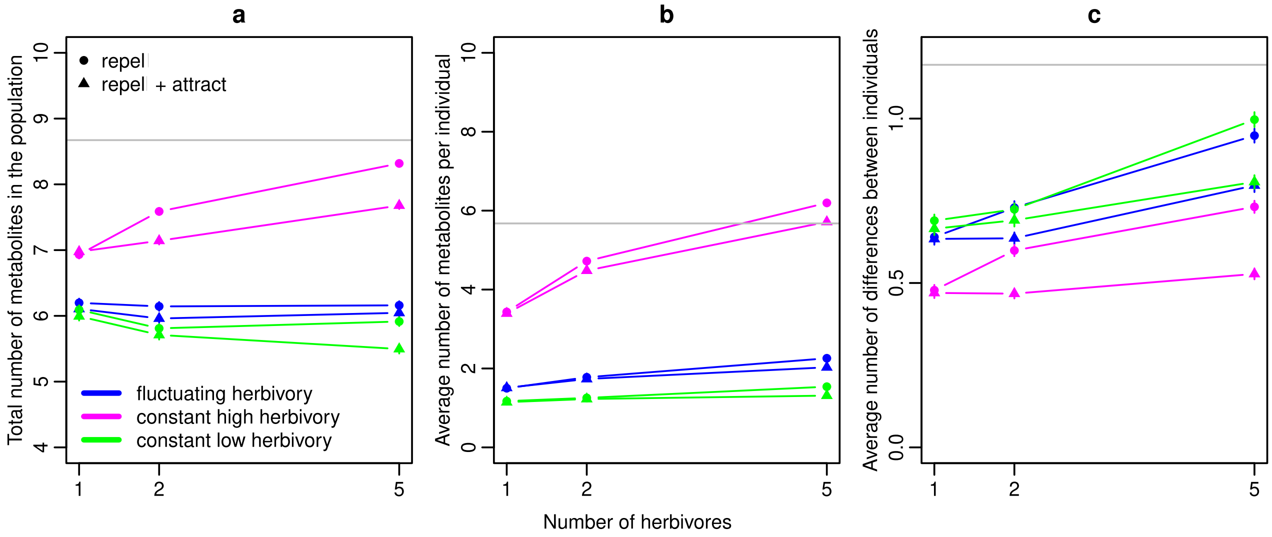

Figure S6 Effect of the number of herbivores and the distribution of effect sizes on chemodiversity patterns when the two dominance coefficients are independently drawn from a uniform distribution on [0,1]. The bars indicate the standard error, but many of them are so small that they fall entirely inside the point and are therefore not visible. The gray horizontal lines indicate average diversity metrics under a neutral model.

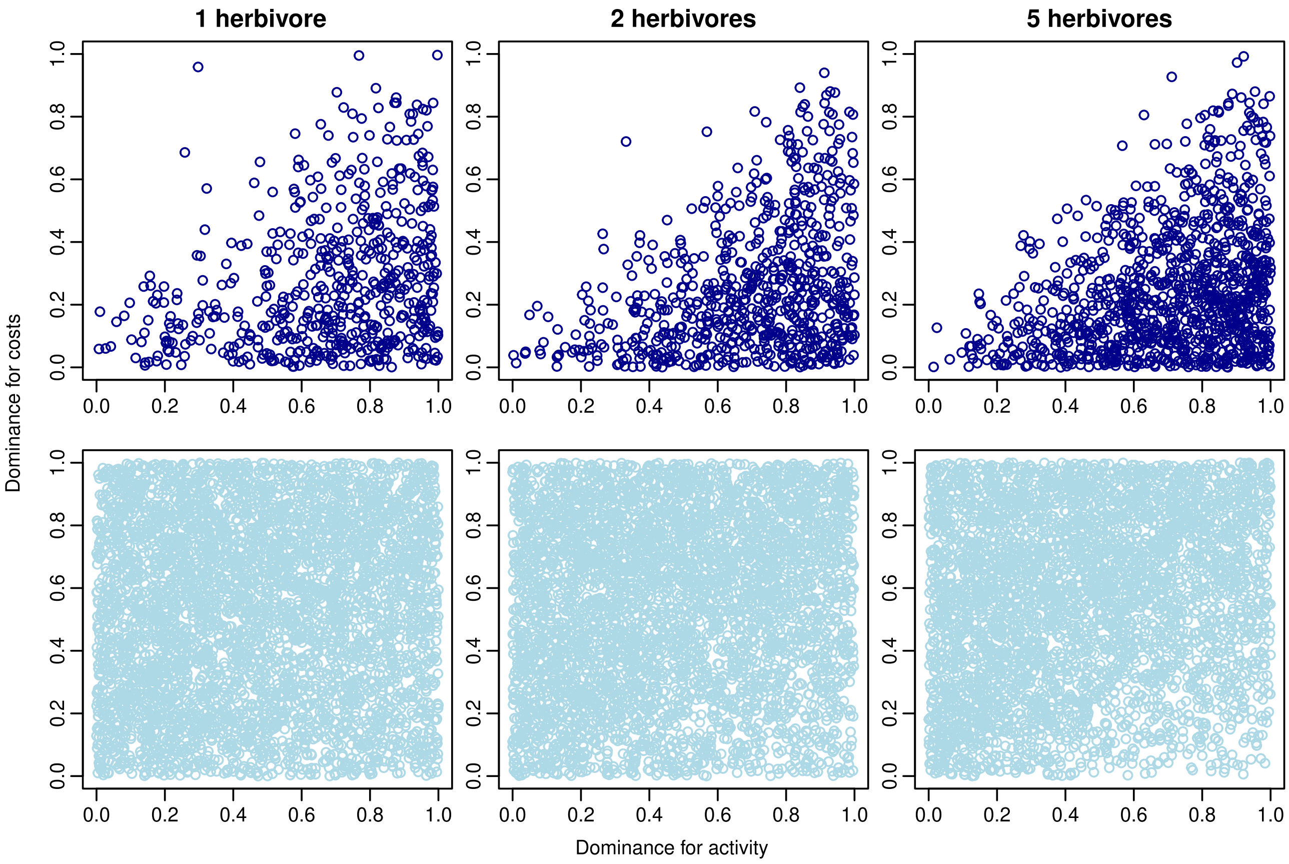

Figure S7 Combinations of dominance for activity and dominance for costs for loci that were polymorphic, i.e., had an allele frequency between 0.1 and 0.9 at the end of the simulation (shown in dark blue in the upper row), and those that were not (shown in light blue in the lower row). The results are for the fluctuating herbivory scenario with only repellent effects of metabolites. The two dominance coefficients were independently drawn from a uniform distribution on [0,1]. Each point shows one locus in one of the 500 replicates.

Additional simulation results for the synergy hypothesis

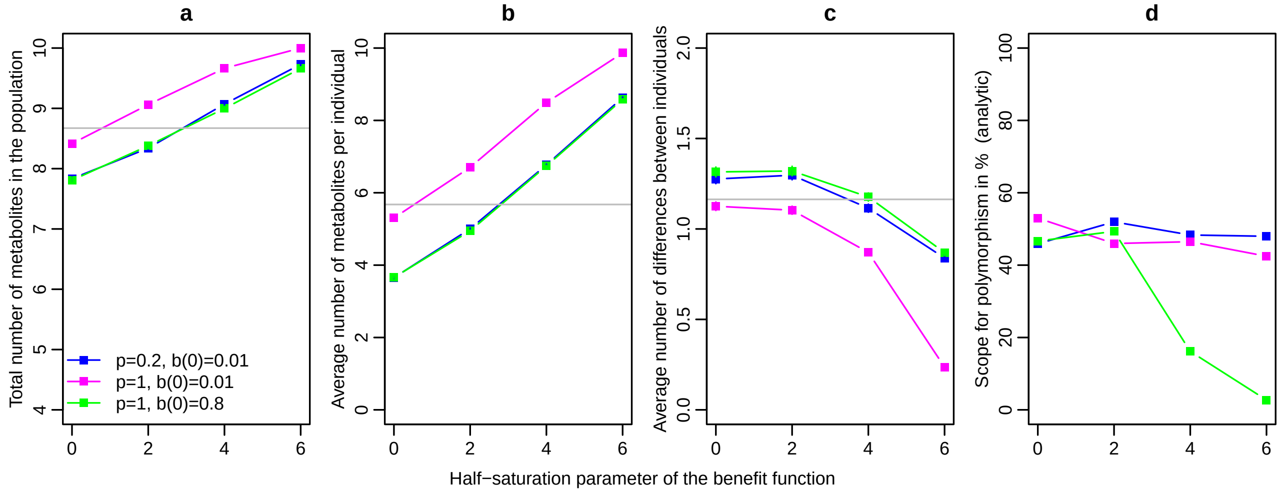

Figure S8 Effect of the shape of the benefit function on patterns of chemodiversity in the case of a single herbivore when the two dominance coefficients are independently drawn from a uniform distribution on [0,1]. With increasing half-saturation parameter, the benefit function becomes more synergistic. The bars indicate the standard error, but many of them are so small that they fall entirely inside the point and are therefore not visible. The gray horizontal lines indicate average diversity metrics under a neutral model.

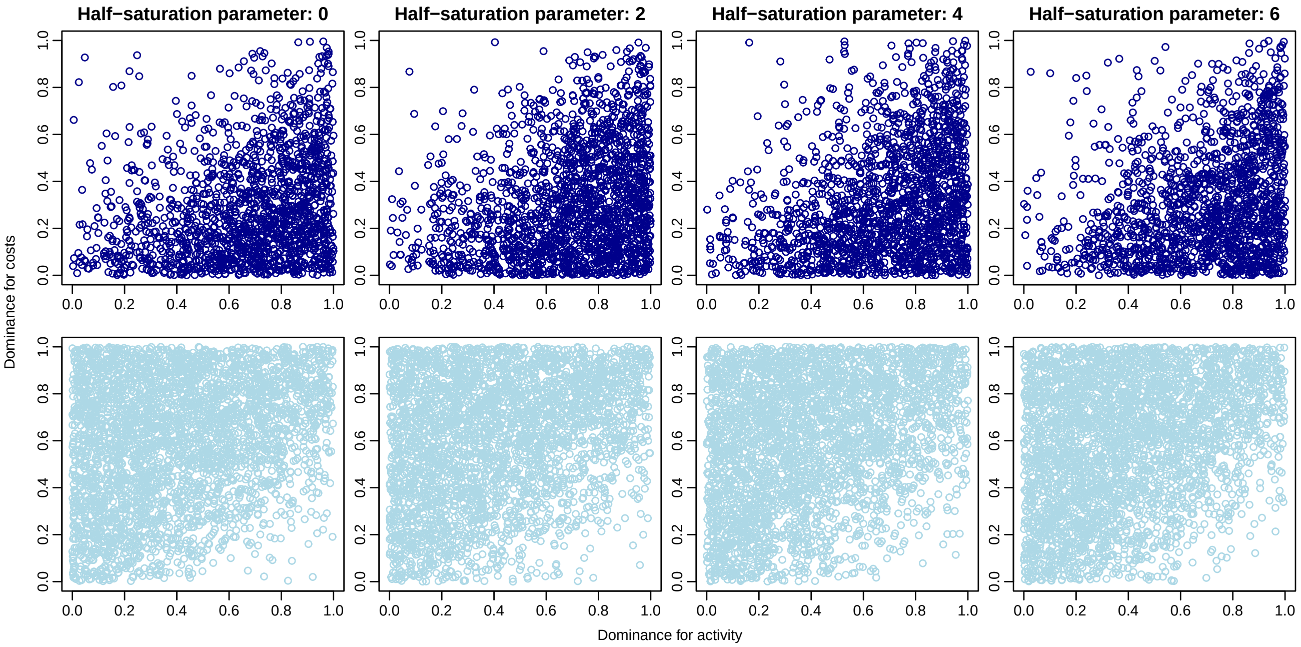

Figure S9 Combinations of dominance for activity and dominance for costs for loci that were polymorphic, i.e., had an allele frequency between 0.1 and 0.9 at the end of the simulation (shown in dark blue in the upper row), and those that were not (shown in light blue in the lower row). The results are for the fluctuating herbivory scenario with one herbivore and $b(0) = 0.01$. The two dominance coefficients were independently drawn from a uniform distribution on [0,1]. Each point shows one locus in one of the 500 replicates.

Notes S3 Conditions for maintenance of polymorphism at a single locus with antagonistic pleiotropy in a constant environment

Here we examine the single-locus antagonistic pleiotropy model by Rose (1982), but parameterized slightly differently so that it becomes more comparable to our model (Table S2). There are two alleles at the locus, 0 and 1. Allele 1 increases fitness component 1, W_1, but decreases fitness component 2, W_2. Like Rose (1982), let us consider the cases of additive selection and multiplicative selection. In each case, polymorphism is maintained if the total fitness ($W_1 + W_2$ in the additive case or W_1W_2 in the multiplicative case) of heterozygotes is higher than that of either homozygote. The dominance combinations for which this is the case are shown in Fig. S10. The regions that allow for polymorphism look qualitatively similar to those in our model. As in our model, there are combinations where polymorphism is possible even though there is no reversal of dominance. It is sufficient if the dominance for the benefits of the 1 allele on W_1 is substantially larger than the dominance for the negative effect of the 1 allele on W_2. The parameter region with polymorphism is largest if selection strength is equal on both fitness components (middle column in Fig. S10). If selection is stronger on fitness component 1, the polymorphism region is reduced to combinations where dominance for fitness component 1 is high. And when selection is stronger on fitness component 2, it is is reduced to combinations where dominance for fitness component 2 is low.

Table S2 Fitness effects in a simple single-locus antagonistic pleiotropy model with two fitness components (W_1 and W_2) that act either additively or multiplicatively.

Genotype	11	10	00
W_1	$1 + s_1$	$1 + d_1s_1$	1
W_2	$1 - s_2$	$1 - d_2s_2$	1
$W_1 + W_2$	$2 + s_1 - s_2$	$2 + d_1s_1 - d_2s_2$	2
W_1W_2	$(1 + s_1)(1 - s_2)$	$(1 + d_1s_1)(1 - d_2s_2)$	1

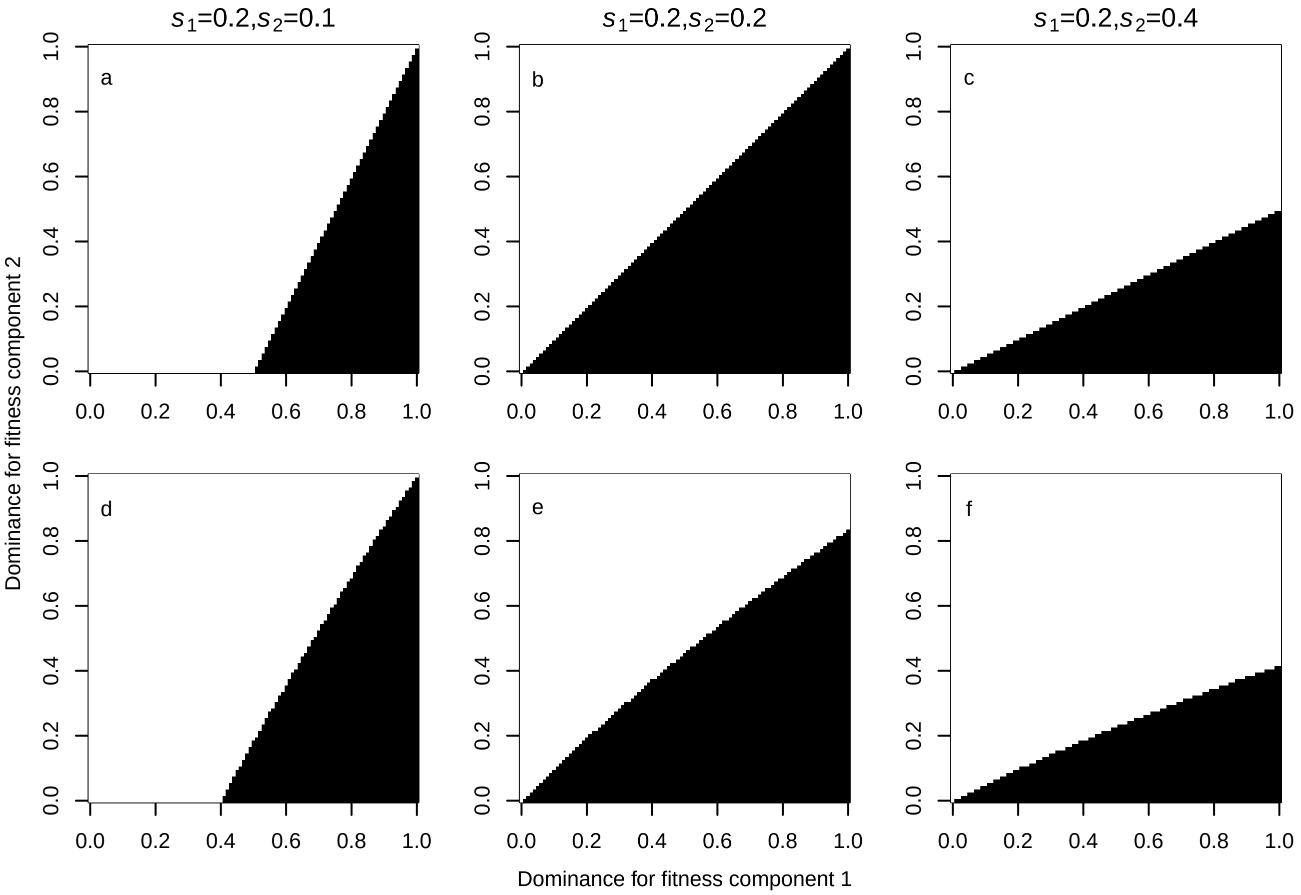

Figure S10 Changes in dominance can maintain polymorphism at a single locus with antagonistic pleiotropy, even without reversal of dominance. The top row shows the region where polymorphism is possible for additive selection and the bottom row for multiplicative selection.

References

Chesson, P. L., and R. R. Warner, 1981 Environmental variability promotes coexistence in lottery competitive-systems. The American Naturalist 117: 923–943.

Rose, M. R., 1982 Antagonistic pleiotropy, dominance, and genetic variation. Heredity 48: 63–78.
